# Understanding intergenerational interactions and programs in Singapore: a comparative analysis of young adults and older adults’ perspectives

**DOI:** 10.3389/fpubh.2026.1774989

**Published:** 2026-03-19

**Authors:** Jasmine Yee Ru Cheng, Audrey Shu Ting Kwan, Wan Qi Yee, Zheng Kwok Sow, Joanne Sze Win Lee, Jamaica Pei Ying Tan, Nigel Teo, Kennedy Yao Yi Ng, Lian Leng Low

**Affiliations:** 1 Division of Population Health and Integrated Care, Singapore General Hospital, Singapore, Singapore; 2 Department of Physiotherapy, Singapore General Hospital, Singapore, Singapore; 3 Oncology and Medicine Academic Clinical Programme, DUKE-NUS Medical School, Singapore, Singapore; 4 TriGen, Singapore, Singapore; 5 Department of Global Health and Population, Harvard T.H. Chan School of Public Health, Boston, MA, United States; 6 Family Medicine Academic Clinical Program, Duke-NUS Medical School, Singapore, Singapore; 7 Research and Translation Innovation Office, SingHealth Community Hospital, Singapore, Singapore

**Keywords:** barriers and facilitators, filial piety, generation gap, intergenerational programs, intergenerational relationships

## Abstract

**Introduction:**

As the population rapidly ages, older adults are faced with a multitude of physical, psychological, and social challenges that limit their ability to age well. Intergenerational programs have gained traction as a potential solution to address social isolation and loneliness among older adults, while also improving intergenerational solidarity among young adults. The study aimed to explore and compare the experiences, facilitators, and barriers to intergenerational relationships and participation in intergenerational programs, from the perspectives of young and older adults in Singapore.

**Methods:**

A qualitative descriptive approach with semi-structured interviews was conducted with 14 young adults and 15 older adults via purposive sampling. Data were analyzed thematically using inductive coding.

**Results:**

Three themes emerged: (1) intergenerational perceptions and attitudes towards intergenerational interactions; (2) language, interpersonal traits, and responsibility to bridge the intergenerational gap as facilitators and barriers towards intergenerational relationships; and (3) the role and impact of intergenerational programs on intergenerational relationships.

**Conclusion:**

This study highlighted the complex interplay of interpersonal, structural, and cultural factors that shape intergenerational relationships and the effectiveness of intergenerational programs in Singapore. To foster authentic and reciprocal intergenerational relationships, intergenerational programs must be intentionally designed to promote equal status, cooperation, and culturally sensitive practices that reflect the lived realities of both young adults and older adults.

## Introduction

The global population has undergone a significant demographic shift. By 2050, the number of older adults, aged 60 years and older, is expected to double from 12 to 24% compared to 2015, reaching 2.1 billion (1). This rapid aging of the population is driven by increasing life expectancy as well as declining birth rate (2). As life expectancy increases, older adults face a multitude of physical, psychological, and social changes that challenge their ability to age well. Notably, social isolation and loneliness have been associated with declines in physical and mental health, cognitive capacity, and life expectancy (3–5). The COVID-19 pandemic further exacerbated these issues: the portrayal of older adults as vulnerable and dependent, along with increased social isolation, has not only heightened intergenerational tensions but also increased young adults’ anxieties about aging (6). These growing concerns about the health and wellbeing of older adults and intergenerational tension have prompted significant interest in ensuring that population ages well.

Singapore is expected to follow a similar trajectory of an aging population; by 2026, the country is projected to become a super-aged society, with at least 21% of the population being 65 years and older (7). Singapore, a multiethnic country in Southeast Asia, influenced by Asian philosophies, such as Confucian ideals, uphold the belief that the family is the fundamental unit of society, with the responsibility of caring for aging parents traditionally falling to the children (8). Notably, filial piety, a set of universal constructs encompassing cultural and moral norms, values, and practices centered around respect and providing care for one’s parents or elders, is a valued belief in Asian communities (9). Within this cultural context, there is a strong emphasis on intergenerational connectedness and mutual support.

As such, intergenerational programs (IGPs), defined as “vehicles for the purposeful and ongoing exchange of resources and learning among younger and older generations for individual and social benefits,” have gained traction as a potential solution (10). A widely applicable theoretical framework underpinning IGPs is the Allport’s Intergroup Contact Theory, which posits that greater intergroup harmony is achieved through four conditions: equal group status, intergroup cooperation, common goals, and institutional support (11). Unlike other frameworks that primarily focus on the dynamics of intergenerational relationships, this theory provides conditions for fostering meaningful interactions between generations, making it particularly relevant in evaluating and understanding IGPs. By bringing together younger and older people, interactions address inaccuracies in individuals’ understanding of other groups. These inaccuracies underlie the formation of stereotypes, and in turn, promote high quality interactions (12). Evidence suggests that IGPs strengthen intergenerational connections and encourage shared experiences, thereby reducing the risk of loneliness and social isolation among older adults (12). Additionally, studies demonstrate that IGPs reduce age stereotyping, increase intergenerational solidarity, and improve self-esteem and empathy among children (< 18 years old) (13, 14). An example is the Tri-Generational HomeCare (TriGen) program, a Singapore student-initiated, intergenerational, interprofessional home visit program where healthcare undergraduates and lay students provide holistic care to community-dwelling older adults (15). An evaluation found that there was a reduction in ageism, development of soft skills, and inculcation of values among volunteers, as well as a reduction in readmission and emergency department visits, and loneliness among older adults (15).

Given these benefits, IGPs have the potential to significantly promote health and wellbeing and intergenerational connectedness among younger and older adults (16–18). Despite the growing literature on the outcomes of IGPs, the experiences and impacts of such programs on young adults (21–35 years old) remain unexplored. Notably, most studies focus on older adults and school-age children (< 18 years old), and little is known about the impact of IGPs on young adults (11). Young adults’ interaction with older adults during this life stage may play a formative role in shaping their perception of aging, enhance empathy, and strengthen intergenerational solidarity (19). Moreover, there remains a gap in understanding how young adults’ experiences compare to those of older adults. A significant body of research has concentrated on understanding older adults’ experiences, and few studies have attempted to compare the impacts of IGPs across both generations (20, 21). However, actively involving young adults in discussions of aging and the development of innovative solutions, such as IGPs, expands the scope of stakeholders and creates more opportunities for meaningful and constructive action (19). Lastly, Singapore’s multicultural, rapidly aging society provides an opportunity to examine these dynamics through both generational and cultural lenses, offering valuable insights into understanding intergenerational relationships as well as providing guidance on the design and delivery of IGPs.

While many studies are guided by established theoretical frameworks, this study took an inductive approach, focusing on participants’ lived experiences. This approach allowed for a more open and nuanced exploration of intergenerational dynamics and programs. As such, this study aims to explore and compare the experiences, facilitators, and barriers to intergenerational relationships and participation in IGPs from the perspectives of young and older adults in Singapore.

## Materials and methods

### Study design

We employed a qualitative descriptive study design with an interpretive approach to explore and compare young adults’ and older adults’ experiences and perceptions of intergenerational interactions and IGPs. This approach was adopted to capture participants’ firsthand experiences and ensure that the findings remained applicable and relevant to the real-world context. The study adhered to the Consolidated Criteria for Reporting Qualitative Research (COREQ) checklist.

### Study site

The study was conducted by the Singapore General Hospital (SGH) Division of Population Health and Integrated Care (PHIC), which oversees care integration initiatives in the Southeast region of Singapore (22).

### Sampling and recruitment

Participants comprised young adults (aged 21 to 35 years old) recruited from various volunteering agencies and volunteer programs in Singapore, such as Youth Corps Singapore, and older adults were recruited from different community care organizations which organized IGPs, among other activities, catering to older adults, such as NTUC Health. They were recruited from different social service agencies and centers in the Southeast region of Singapore who are regular partners of SGH. Participants were recruited through purposive sampling to ensure inclusion of varied background and perspectives. We recruited participants through a research recruitment poster, which was circulated to several organizations for recruitment. All participants must have taken part in at least one IGP, involving both age groups, within the last three years.

Participants who were interested in taking part in the study provided their contact information through an online form or via telephone to a study team member through the contact number provided on the publicity poster. Subsequently, an interview, conducted in either English, Mandarin, or Malay, was scheduled with participants. Signed consent was obtained from participants after the study information was shared, all questions were addressed, and they were reassured that they could skip questions or withdraw from the study at any point in time. We continued recruitment until we determined that data saturation had been achieved, when no additional information arose from the interviews.

### Data collection

The interview guide was developed based on topics related to intergenerational relationships and IGPs. A summary of the interview guide is presented in Table 1.

**Table 1:** Summary of interview guide.

Topic	Sample interview questions
Participating in IGPs	Can you share more about the IGP you attended?
Interactions with Older Adults/Young Adults	Can you share your experiences interacting with older adults/young adults through the program(s) you participated in? In your experience, was it easy or difficult to interact with older adults/young adults? How do you think we can better encourage young adults/older adults, like yourself, to join IGPs? And what is the most important factor?
Friendship with Older Adults/Young Adults	What is your experience in forming friendships with older adults/young adults like?
Generation Gap	Is the generation gap something you have experienced with older adults/young adults? How do you think older adults/young adults view you?
Suggestions for IGPs	If you come up with a program to bring older adults and young adults in the community together/to encourage friendships between older adults and young adults, what will this look like?

Five interviewers (WQ, ZK, JT, JL, NT) trained in qualitative data collection methods conducted one-to-one semi-structured interviews with participants. Interviews were conducted via Zoom Web Conferencing (23) or in-person, with each interview lasting between 60 to 90 min. During each interview, one study team member was the main interviewer, and the second team member was a moderator by taking key notes, observing the participants’ tone and expression, and asking further questions when necessary. All interviews were audio-recorded and transcribed verbatim by either a student intern, a research team member, or a third-party transcription service. All transcripts were verified by a study team member to ensure verbatim accuracy and redact identifiable information. All data were de-identified and stored on a set of electronic devices accessible only by the research team.

### Data analysis

Five team members (JC, WQ, NT, JT, AK) coded the transcripts using an inductive approach. Based on Braun and Clarke’s six-step framework, thematic analysis was utilized to analyze the transcripts (24). QSR NVivo 14 Software was used for data management and analysis.

Each transcript was independently coded by two of the five investigators and then checked by a third to address any discrepancies. Codes were combined iteratively to form themes through discussion with the research team. Final themes were refined and named to ensure they adequately represented the data findings. We enhanced the trustworthiness of our study through three strategies: (i) maintaining methodological transparency and alignment with our research question to ensure credibility, (ii) implementing double-coding and inclusive data representation to ensure confirmability, and (iii) relating our findings to the literature to ensure transferability.

### Research team and reflexivity

All research team members were affiliated with the Division of PHIC at SGH and were public health researchers. The team had prior experience with implementing programs with intergenerational elements but did not hold any authority over the participants. Before the study, the research team briefed participants on the interview process and ensured that they were in a comfortable environment. The research team also encouraged participants to share their responses openly and honestly, assuring them that there would be no judgment.

To enhance reflexivity during data collection, interviewers engaged in pre- and post-interview debriefing to reflect on how our perspectives might influence questioning and interpretation. The semi-structured interview guides increased consistency, and several investigators cross-checked responses to highlight and consider diverse perspectives. Given our institutional position, we were conscious of power dynamics. As such, to encourage candid responses, participants were reassured that their perspectives would be received openly and without judgment. To mitigate potential biases, we critically examined our positionality and its potential impact on data collection, analysis, and write-up.

### Ethics statement

Ethics approval was granted by the SingHealth Centralized Institutional Review Board (Reference No. 2021/2773).

## Results

A total of 14 young adults and 15 older adults were recruited and interviewed between January to July 2022. The nature and characteristics of the programs young adults participated in were included in Table 2. However, a majority of older adults did not remember the exact IGPs they participated in. Participants only provided brief descriptions of them which varied in duration and outcomes. Typically, young adults took on facilitator roles while older adults participated as beneficiaries in recreational activities, like arts and crafts and befriending. Moreover, not all participants provided the frequency of their participation in IGPs; “ad-hoc” refers to participants who have participated in a program once, while ‘multiple sessions’ refers to participants who have participated in a program at least twice.

**Table 2:** Summary of characteristics of young adults and older adults.

Young adults (*n* = 14)	
Sex	
Male	6 (42.9%)
Female	8 (57.1%)
Age	
Mean age	25
Age range	21–35
Race	
Chinese	12 (86.7%)
Malay	2 (14.3%)
Young adults; IGP characteristics	
Duration of IGPs	
Long-term	10 (71.4%)
Multiple	4 (28.6%)
Nature of IGPs	
Befriending	5 (35.7%)
Health and exercise	5 (35.7%)
Digital	2 (14.3%)
Interest Group	2 (14.3%)

Older adults (*n* = 15)	
Sex	
Male	4 (26.7%)
Female	11 (73.3%)
Age	
Mean age	77
Age range	54–89
Race	
Chinese	11 (73.3%)
Malay	4 (26.7%)

The findings were organized into three themes: (1) views and attitudes towards intergenerational interactions, (2) facilitators and barriers towards intergenerational interactions, and (3) the role and impact of IGPs.

### Theme 1: intergenerational perceptions and attitudes towards intergenerational interactions

This theme explored how participants viewed intergenerational interactions, which were shaped by a mix of personal values, cultural expectations, and prior experiences. Many participants expressed an openness to engage those from different age groups. Notably, older adults reported that young adults were respectful and polite to them, and that most of their interactions was sincere and friendly.

*“They are very good; they treat us with a lot of respect. We really appreciate them.” (OA006)*

Older adults also reported that young adults were caring and considerate of their interests and needs. For instance, older adults reported that young adults were patient in explaining to them unfamiliar concepts like digital technology.

*“When we were a bit slow to learn things, the young people would say, ‘Ah Ma, do not worry, we will teach you’… made us have a peaceful mind that they teach us, and we did not need to be scared.” (OA001)*

Young adults similarly reported positive perceptions of older adults. They reported that older adults were understanding of the challenges and struggles they faced. They appreciated older adults’ generosity and wisdom in sharing their life experiences and advice with them.

*“… they have experienced a lot more…so there’s a lot of things that they say that [are] true to their words.” (YA008)*

These positive interactions encouraged them to engage with individuals from the other generation. For instance, one participant reported that they formed close relationships after learning that they had shared interests and hobbies with older adults.

*“… I think after a while I realized that there can be different ways of communicating with [older adults], so maybe through actions or through shared interests, like for example, food or hobbies…” (YA004)*

However, some participants expressed some negative views of each other. They expressed frustration and reluctance towards interacting with individuals from the other generation. Young adults reported feeling dismissed by older adults’ belief that they were more knowledgeable and experienced than young adults.

*“… [older adults] like to voice their opinions…they’re always like, ‘oh, listen to me, because we know more’… doesn’t mean you’re older, you get more like consideration from other people or like your opinions matter the most.” (YA012)*

Young adults reported that this was exacerbated by older adults’ persistent mindset, which augmented these feelings of being overlooked. As a result, young adults preferred to limit their interactions or placate older adults by agreeing with what they say, even if they do not agree with them. One young adult highlighted:

*“It’s almost no point in you putting so much effort because they are fixated on a certain thought or mindset. So, if you keep, like, trying to do anything about it, it’s just going be one-sided…my consensus is just suck it up, just keep nodding your head, then when [they’re] done talking, eventually you can just proceed with your life.” (YA008)*

Likewise, some older adults reported that young adults appeared to interact with them out of respect, rather than from an interest in knowing them better. Some older adults also voiced their unhappiness towards young adults’ rude and disrespectful attitudes, especially when older adults attempted to give well-meaning advice to them.

*“But we can teach them and warn them, ‘don’t be so short-tempered, don’t be so angry…you should treat us with respect, cannot like that,’ but they will also just laugh it off.” (OA003)*

Additionally, older adults observed young adults to be impatient and inconsiderate. For example, young adults became annoyed when older adults were slow to learn new things, and scolded them, “so stupid…you! Learn already, still don’t know,” which older adults reported feeling hurt and frustrated.

### Theme 2: facilitators and barriers towards intergenerational relationship

This theme explored the factors that influenced the development and quality of intergenerational relationships. These included language, interpersonal traits, shared interest and cultural norms, and willingness to form intergenerational relationship.

#### Theme 2.1: language as a bridge and barrier

Participants identified language as a key facilitator in the development of intergenerational relationships. In Singapore, young adults are accustomed to speaking English, while older adults are often more comfortable speaking their Mother Tongues (e.g., Chinese, Malay, and Tamil) and dialects (e.g., Hokkien, Teochew, etc.). Participants reported that if both generations were able to converse in the same language, they were able to “build rapport and trust.” One participant described how learning a dialect improved their relationship with older adults.

*“[Program name] actually provided like for example…short language sessions…you kind of have a better understanding and better communication with the [older adults] as well…I’m slightly more confident in terms of engaging the [older adults] and look forward to meeting them…” (YA004)*

This effort to become more proficient in a language was perceived as a sign of respect and genuine interest in engaging and knowing older adults better.

Conversely, participants who were not fluent in a language, especially young adults, encountered challenges when engaging older adults. Participants reported difficulty comfortably communicating with each other.

*“…when I don’t really know most of the words in dialect, I think the [older adults] will actually switch to Mandarin. I think the switching part right…it makes the conversation not as genuine and not as authentic as what it can be when we actually use [dialect] as a way of communication.” (YA007)*

One older adult reported that when young adults communicated in English, they felt it was “not as friendly as [mother tongue language],” as conversations were not as intimate compared to conversations spoken in the same language.

#### Theme 2.2: lack of common topics and cultural disconnect as a barrier

While having a common language was valuable in improving rapport between the two generations, participants reported that they faced difficulties talking to participants when there was a lack of common topics. This proved to be a challenge when participants found it difficult to relate to each other, thus making it difficult to sustain and engage in meaningful conversations.

Older adults noted that it was difficult to understand young adults’ mindset and chose to interact with them less. Older adults shared that intergenerational differences in values or priorities made it harder to find common ground.

*“[Young adults] have their own mentality. Our thinking is ‘take the days as they come.’ Different ways of thinking.” (OA006)*

Aside from a lack of common topics, older adults reported that observance of cultural values and traditions was an area of disconnect. For instance, observance of traditional festivals, such as *Qing Ming*, a day to remember and honor the deceased through cleaning gravesites and performing ritual offerings to their ancestors, was often celebrated by older adults and rarely by the younger generation.

Moreover, participants cited that having different hobbies and interests made it difficult for both parties to engage in meaningful conversations. Conversations about sensitive topics, such as marriage, career, and politics, often led to disagreements and tensions due to different beliefs that each generation holds.

*“I think in certain aspects, there is a certain gap…like, maybe if I were to converse with them about—what are their opinions on education, or politics…then there might be a gap in terms of their opinions.” (YA004)*

As a result, individuals from both generations avoided discussing these topics for fear of worsening relationships.

*“If we say too much, people will say that we are nosy. People don’t want to discuss such topics. For example, if a young person were to hear the word ‘marriage’ from a grandmother, they would be uncomfortable.” (OA006)*

#### Theme 2.3: relational traits that foster connection as a facilitator

Participants also reported various traits that facilitated intergenerational relationships. Firstly, older adults were appreciative of young adults who were respectful towards them. Likewise, young adults reported that being respectful and understanding facilitated relationship building. As a result, older adults were more willing to open up and engage in conversations. For example, if older adults were short-tempered or less trusting towards them, young adults would attempt to reassure them, which helped to create more genuine conversations and trusting relationships in the future.

*“I think they’ve been through a lot of different friendships and other difficulties that sometimes they just don’t open up, maybe because of betrayal or something…so I think we really need to show that, ‘hey, I’m here to build a bond with you and be here regularly’, then they will start to trust you…” (YA004)*

Older adults also highlighted that being open to learning and interacting with young adults facilitated interactions. One older adult reported how interacting with young adults was vital towards dispelling stereotypes of older adults.

*“Some people said, ‘we are dirty, people do not like to talk to us.’ I said, ‘no lah, you do not talk to people, then the people will not talk to you, right?’… [young adults] really do not have good exposure to what the real old people look like in public.” (OA001)*

Some participants maintained an open mind about these differences and embraced different perspectives. Participants acknowledged that as societal norms continue to evolve, it is imperative to be aware and sensitive to these changes. An older adult illustrated how understanding and accepting the different parenting styles of each generation allowed her to maintain better relationships with her children and grandchildren.

*“Today’s children are not like those in the past, parents are like friends with their children, and then families will be happy. If you are strict and overbearing, the child will rebel and do the opposite.” (OA003)*

Lastly, older adults reported that they viewed young adults as companions, emphasizing the importance of being kind, patient, and friendly as they would treat a friend. Despite coming from different generations, they believed that both older adults and young adults share a lot in common. Participants noted that people from both generations shared fundamental values that transcended generational boundaries.

*“I don’t think the difference is coming [from] the different [generations]… it’s subjective to individual persons…different people have different backgrounds…certain values or philosophies that the [older adults] have, I would have some as well. So, it’s really about the person’s character as well as their background…” (YA004)*

#### Theme 2.4: perceived role to form intergenerational relationship

A small group of participants reported differing views on willingness towards forming relationships with individuals from the other generation. Among young adults, they reported a sense of personal and generational responsibility to initiate connections with older adults. They viewed their position as being younger, and thus more socially adaptable, technologically savvy, or culturally aware, as their responsibility to better understand older adults. One participant articulated this belief:

*“Like anything, it should be teaching the young adults to address the generation gap…” (YA010)*

Similarly, some older adults believe that young adults should be the one to initiate in forming relationship with them. They reported that the onus should be on young adults to take the initiative, not only because of their capabilities or energy, but also as a reflection of social values of filial piety:

*“Doing activities with the youth…the youth should help the [older adults] out more. Otherwise, if it’s the [older adults] helping [young adults] more, then it’s not right.” (OA003)*

### Theme 3: the role and impact of intergenerational programs on intergenerational relationships

This theme explored the benefits of IGPs in facilitating intergenerational relationships, implementation challenges, and suggestions for improvement.

#### Theme 3.1: benefits received by young adults and older adults after participating in intergenerational programs

Young adults reported that through IGPs, they had the opportunity to listen to older adults’ experiences and life advice, which many were grateful for.

*“… when they share their life stories, I think this is something that can really add to my own experiences. Sometimes, we don’t have to go through, then just by hearing their experiences, right, you get to learn without going through…” (YA011)*

Young adults also reported that they developed soft skills, such as active listening and empathy, when engaging with older adults. One participant recounted her experience interacting with an older adult who was unwilling to take part in an activity,

*“… [older adults] say we shouldn’t take [their actions] to heart…I think there were a few times we got scolded by the [older adult], because she just doesn’t want to do [the exercise]… and it affected our morale, but after I think for a while, we realised that…maybe she doesn’t know what she’s doing…so we don’t take it to heart.” (YA004)*

Many young adults also reported feeling closer to their community. They took the initiative to look out for and offer help to older adults when they could. One young adult reported that even after the IGP had ended, she would visit the older adult every 2 months to check in on her. These regular check-ins eventually developed into a friendship.

Moreover, instead of doing individual activities, participating in activities with other older adults, or staying at home, older adults reported that they enjoyed being able to participate in IGPs with young adults. Older adults reported that IGPs helped them to pass the time quickly. As most of the older adults were retired, they had the free time but not many activities to fill it. Older adults were also not concerned with the type of activities offered but were just happy to participate alongside young adults. Seeing young adults enjoying themselves made older adults happy as well.

*“As long as the youths come, we would all be happy. Most importantly, we see that [young adults] are cheerful and happy.” (OA006)*

### Theme 3.2: implementation challenges of intergenerational programs

Firstly, participants highlighted the difficulties of committing to long-term programs. Young adults noted that with other competing priorities, it is often difficult to commit to regular long-term programs.

*“… I do have other considerations as well, like, for example, like to meet up with friends, or families, and even like really have some self-care time, like for myself, to really do, like, my hobbies, and what I really like.” (YA007)*

Older adults also cited reasons such as having to look after grandchildren, going for medical appointments, and unpredictable health concerns as barriers to participating in long-term programs.

Secondly, participants reported that while IGPs provided opportunities for shared activities, there were limited opportunities for meaningful opportunities. Many IGPs were designed such that young adults took on the role of facilitators, while older adults were positioned primarily as beneficiaries. This often resulted in one-way interactions without reciprocity or relationship-building. Several young adults highlighted that need for IGPs to create space and time for open conversation and mutual learning. One young adult cited that unstructured space and time after an IGP helped build rapport.

*“It’s supposed to be a therapeutic event, become like some speed test leh…[program organizers] give us full three hours…people finish in two and a half hours…then [older adults] sit back and they chat with us…just that space and time brings out so much difference.” (YA006)*

Thirdly, participants reported that IGPs’ activities which required specific knowledge or skills were often not suitable or inclusive. For instance, *mahjong,* a four-player tile-based strategy game, often excluded young participants as there may not be enough players or they may not be familiar with the game. Older adults also reported that there were activities which were not suitable for those with limited mobility or dexterity, which were frustrating for those with limited physical abilities. Moreover, older adults faced difficulties with learning new activities. One participant expressed their dejection after not being able to learn the activity.

*“And I was quite stupid, thinking, and learning things slowly. I did not understand [the instructions]. So, it will be miserable.” (OA013)*

### Theme 3.3: recommendations to improve intergenerational interactions and programs

Firstly, participants prioritize flexibility. While young adults recognize the disadvantages of short-term volunteering, they also believe that requiring participants to commit to a long-term program puts undue pressure to partake in IGPs, which may deter participants from participating in the future. One participant proposed,

*“… let it be ad-hoc volunteering, then they might feel that ‘oh, there is something for me,’ then they will actually continue to join in future sessions, yeah, maybe on a more regular basis.” (YA007)*

Secondly, young adults suggested having activities where participants from both generations can participate together on equal footing and in an inclusive manner.

*“… when it comes to activities, it’s always nice to have to do things whereby we’re on an equal level…doesn’t mean that the junior will do better than the [older adult] or the [older adult] will do better than the junior, that kind of activities whereby if they’re on the same level…wouldn’t have the message of, ‘oh, this activity the junior will definitely do better’, that sort of thing.” (YA011)*

Thirdly, as young adults often feel inexperienced in interacting with older adults, young adults suggested providing support and guidance for inexperienced volunteers by including guiding questions or having experienced volunteers present. Older adults also recommended educating young adults on how they should treat older adults. A participant highlighted that young adults might not understand the circumstances of older adults and judge them too quickly.

*“If you suddenly ask them to see old people, they will think why the old people are like that. You need to explain to them that the old people did not have a person to look after them.” (OA001)*

Many young adults echoed this sentiment and recommended that participating in IGPs be made mandatory from a young age. They also recommended increasing the presence of older adults-related content on social media to increase young adults’ exposure to and understanding of older adults.

*“So, I think for a start, for people who have not been exposed to volunteer at all, it would be good to make them do it lah, and how do we make them do it, is by making it compulsory…once we are able to expose the younger people who are still in the public education system to do it, then it will become easier for them to carry on with it as they move to like [university] or like their young adult working life.” (YA010)*

## Discussion

This qualitative study explored and compared young adults’ and older adults’ experiences, facilitators, and barriers of intergenerational relationships and IGPs. Specifically, we sought to understand how each generation perceives each other, the factors that enable or hinder meaningful intergenerational interactions, and the perceived role and impact of IGPs. These findings are interpreted through Allport’s Intergroup Contact Theory, which identifies four conditions necessary for positive intergroup contact: equal status between groups, common goals, intergroup cooperation, and institutional support. Our findings revealed how Singapore’s hierarchical cultural context systematically undermines equal status cooperation, while language barrier and program design compromise cooperation and achievement of common goals.

Our results revealed positive perceptions of intergenerational interactions, with both young adults and older adults valuing mutual respect, patience, and shared experiences. However, both groups expressed frustration as a result of perceived hierarchical dynamics and communication gaps. In the Singapore context, where cultural norms emphasize deference to elders, young adults cited feelings of obligation to conform to older adults’ authority, while older adults felt that their status was being challenged. In hierarchical cultures, like Singapore, cultural norms and practices often reinforce unequal power dynamics, leading to justification and acceptance of hierarchical relationships (25). This dynamic can undermine the equal status condition, which is vital for reducing intergroup prejudice and fostering meaningful relationships. This was corroborated by a cross-cultural meta-analysis which found that individuals in more egalitarian societies demonstrated less prejudice in intergroup contact compared to more hierarchical societies (26). These findings demonstrated the importance of understanding intergenerational relationships within their cultural context.

The results also highlighted various facilitators and barriers that influenced the development and quality of intergenerational relationships, directly corresponding to factors that either supported or undermined the cooperation and achievement of common goals conditions of effective intergroup contact. In a multi-linguistic environment like Singapore, language emerged as both a facilitator and a barrier in intergenerational interactions. Give that communication is foundational to meaningful social connections and mutual understanding, language barriers directly undermine the cooperation and achievement of common goals conditions (27). When participants are unable to communicate effectively, they struggle to identify shared objectives or engage in collaborative activities that could foster shared identity and emotional connection. This was supported by a study which examined the evolution of IGPs in Singapore and found language to be a significant facilitator and barrier among both young adults and older adults, where fluency in a spoken language either increased or limited opportunities for relationship building (28). While language had the ability to ease initial interactions, participants often noted that meaningful interactions were hindered by a lack of common topics. Differences in interests and values limited meaningful engagement with participants often avoiding communications for fear of conflict. In a hierarchical society like Singapore, individuals often find interpersonal conflict distressing and avoid such conflicts to preserve interpersonal harmony (29). Despite these barriers, several constructs emerged as facilitators of intergenerational relationships. A scoping review of Roger’s person-centered approach identified constructs which are vital in facilitating intergenerational relationships, of which congruence, warmth, empathy, unconditional positive regard (UPR) was observed in our findings (30). For example, young adults who reassured older adults, and showed consistency in their efforts helped foster more open and trusting relationships, and older adults’ willingness to open up and share their experience, reflected warmth and UPR. Similarly, when young adults recognized that older adults might be guarded due to previous negative experiences with young adults, or when older adults acknowledged that young adults may hold misconceptions about aging, they demonstrated empathy. Lastly, when participants expressed a genuine interest in building bonds, such as openness to different perspectives like different communication approaches, it was characteristic of congruence.

Building on these facilitators and barriers, our results also revealed that young adults perceived themselves to be responsible for building relationships with older adults. This was evident in young adults’ sense of personal and generational responsibility, which was echoed by older adults. This sense of responsibility was deeply influenced by filial piety. Drawing on Yeh and Bedford (31) dual filial piety model, participants’ behaviors and attitudes reflected both reciprocal and authoritarian dimensions. Our results revealed that young adults described a genuine affection and desire to care for older adults, consistent with reciprocal filial piety. Simultaneously, many also expressed a sense of duty or pressure to show deference and respect, even in challenging interactions, which was indicative of authoritarian filial piety. Li et al. (9) found that Singaporean participants scored significantly higher on the authoritarian filial piety sub-scale compared to their Australian. Our findings demonstrated that both generations valued the respect and care young adults provided, reflecting how cultural expectations about young adults’ roles shaped these relationships. This dynamic creates a paradox within Allport’s Intergroup Contact Theory: while young adults’ caring behaviors might appear to facilitate positive contact, the underlying power imbalance violates the equal status condition. This authoritarian dimension of filial piety makes it difficult to achieve balanced relationships. This effect may be compounded by older adults’ preference for casual interactions over deeper intergenerational relationships, who prioritize meaningful and familiar relationships over novel and effortful ones (32).

Our study also highlighted that aside from providing opportunities for young adults and older adults to interact, IGPs provided valuable opportunities for young adults to gain life insights and develop important soft skills, such as empathy and communication skills. This aligned with previous research that younger participant developed similar soft skills (31, 33), as well as an appreciation of live advice from older adults, especially when it is perceived as constructive (34). Additionally, older adults reported that they valued the sense of community forged through IGPs, with young adults similarly reporting a greater sense of interpersonal support and belonging. However, our results revealed that older adults prioritized young adults’ enjoyment over their own. This finding aligned with Chong and Liu’s finding that older adults in contemporary society may hold lower expectations of filial piety but derive greater satisfaction and wellbeing from giving, such as showing concern for young adults (35). However, this dynamic may unintentionally undermine the condition of cooperation. When older adults assume a passive role to elevate young adults’ experiences, opportunities for mutual collaboration and shared purpose may be reduced. This imbalance can reinforce hierarchical dynamics and prevent the facilitation of intergenerational relationships on equal terms.

Despite their benefits, IGPs faced several implementation challenges. Firstly, participants highlighted difficulties with long-term commitments due to competing priorities such as work, studies, or caregiving responsibilities. This was echoed in Jarott (36) study, which identified scheduling conflicts and having multiple commitments as reasons for participant attrition from programs, thus limiting long-term relationship building. Secondly, participants reported limited opportunities for reciprocal interactions. Many IGPs were structured with young adults as facilitators and older adults as beneficiaries. This asymmetrical structure reinforces a power dynamic and undermines the reciprocity needed for successful intergenerational relationships (37, 38). Such a structure not only contradicts the equal status and cooperation condition, but reinforces authoritarian filial piety, where young adults are the active agents while older adults are respected but passive figures. This prevents genuine cooperation by limiting opportunities for collaboration and focuses on service delivery as opposed to ensuring mutual benefits. Only institutional support is achieved, but this alone is insufficient for positive intergroup contact, which can unintentionally hinder authentic intergenerational interactions. Thirdly, program activities perceived as unsuitable or favoring one generation often led to reduced participation and engagement. Maulod et al. (4) similarly highlighted that programs that fail to accommodate the diverse needs, abilities, and interests of their participants risk marginalizing participants and diminishing their sense of belonging. Collectively, these challenges highlight the need for IGPs to adopt culturally sensitive, reciprocal, and structurally inclusive designs that uphold the core conditions of effective intergroup contact.

### Recommendation for future IGPs

Based on our findings, several suggestions are recommended to enhance the quality of IGPs, as described in the following subsections.

### Incorporating flexibility into IGPs

Flexibility was highlighted as a crucial consideration. Maulod et al. (4) recommended incorporating flexibility into the program while not compromising participants’ capability to form intergenerational relationships, such as having befriending frequency matched to older adults’ needs and young adults’ commitment. This approach supports consistent interactions and ensures that interactions remain sustainable and meaningful, while supporting the evolving needs of its participants.

### Promoting equal status within IGPs

Participants emphasized the importance of activities that positioned both age groups on equal footing. Kende et al. (25) found that even in hierarchical cultures, having equal and balanced interactions reduced prejudice compared to unbalanced ones, reasserting that intergroup interactions are more effective when participants perceive equal status and cooperate toward common goals. Activities that promote shared learning, mutual engagement, and joint goal setting were more likely to nurture intergenerational understanding and respect, as well as facilitate intergenerational relationships.

### Ensuring structured support for participants

Our findings revealed limited evidence of systematic institutional support. However, young adults recommended providing structured guidance for new volunteers, such as including suggested questions or having seasoned participants mentor volunteers. Mentorship and supervision were found to be essential in ensuring the quality and motivation of volunteers.

Participants also strongly advocated to incorporate educational components that promote awareness of the ageing process and its associated challenges. Having structured exposure provides participants opportunities to learn about age-related changes, stereotypes, and common barriers faced by older adults (39). This knowledge helps foster empathy, reduce misconceptions, and better prepare young adults for meaningful engagements. These recommendations align with Allport’s Intergroup Contact Theory’s institutional support condition, suggesting that effective intergenerational contact requires not just program availability, but structured institutional frameworks that prepare participants and create conditions conducive for positive interactions.”

### Strengths and limitations

There are several strengths to this study. The use of an interpretive approach provided insight into intergenerational interactions and IGPs through a local lens. The interaction of a common intergenerational theory and culturally specific norms provided further depth into intergenerational relationships and the importance of the structure of IGPs. Additionally, including both young adults and older adults’ experiences and perceptions contributed to a more comprehensive understanding of intergenerational interactions and IGPs in Singapore.

Nevertheless, there were some limitations. Firstly, participants attended IGPs during the height of COVID-19 pandemic when there were restrictions on in-person interactions. At the point of the interviews, most of the restrictions had been lifted, as such, the findings may not be applicable to current IGPs. Nonetheless, the findings provided valuable insight into how participants responded to challenges during such an unprecedented time, highlighting the resilience of intergenerational connections and the importance of the design of IGPs. Secondly, while the interpretive approach provided new insights into intergenerational interactions, not all of them were explored in-depth. For instance, the sense of responsibility felt by young adults could have been further explored to understand its impact on their interaction with older adults. Thirdly, the inclusion criteria comprised individuals who have participated in an IGP within the last three years, making participants susceptible to recall bias, as compared to interviewing participants at the end of IGPs. Lastly, the authors did not collect data on the nature of IGPs (i.e., types of IGPs, facilitation structure, intensity, and types of IGPs, etc.) and other contextual information (e.g., older adults’ health status) which could have provided further depth to the analysis of participants’ experiences.

## Conclusion

In conclusion, this study highlighted the complex interplay of interpersonal, structural, and cultural factors that shape intergenerational relationships and the effectiveness of IGPs in Singapore. While Allport’s Intergroup Contact Theory served as a valuable framework, conditions like equal status and cooperation were often challenged by age-based hierarchies and filial piety. These cultural norms, when unaddressed in program design, may reinforce power imbalances and hinder meaningful engagement. To realize the full potential of IGPs, programs must be intentionally designed to promote equal status, cooperation, and cultural sensitivity.

## Data Availability

The original contributions presented in the study are included in the article/Supplementary material, further inquiries can be directed to the corresponding author.
