# Supplementary material for: Understanding intergenerational interactions and programs in Singapore: a comparative analysis of young adults and older adults’ perspectives
Source: Front Public Health. 2026 Mar 19;14:1774989. doi: 10.3389/fpubh.2026.1774989 (PMC13045879; doi:10.3389/fpubh.2026.1774989)
Supplement: Supplementary file 1 [file Table_1.docx]

**COREQ (COnsolidated criteria for REporting Qualitative Research) Checklist**

| **Topic** | **Item No.** | **Guided Questions/Descriptions** | **Reported on Page No.** |
| --- | --- | --- | --- |
| **Domain 1: Research team and reflexivity** | | | |
| Personal characteristics | | | |
| Interview/facilitator | 1 | Which author/s conducted the interview or focus group? | Materials & Methods |
| Credentials | 2 | What were the researcher’s credentials? E.g., PhD, MD | Materials & Methods |
| Occupation | 3 | What was their occupation at the time of the study? | Materials & Methods |
| Gender | 4 | Was the researcher male or female? | NA |
| Experience and training | 5 | What experience or training did the researcher have? | Materials & Methods |
| Relationship with participants | | | |
| Participant established | 6 | Was a relationship established prior to study commencement? | Materials & Methods |
| Participant knowledge of the interviewer | 7 | What did the participants know about the researcher? E.g., personal goals, reasons for doing the research | Materials & Methods |
| Interviewer characteristics | 8 | What characteristics were reported about the interviewer/facilitator? E.g., Bias assumption, reasons and interests in the research topic | Materials & Methods |
| **Domain 2: Study design** | | | |
| Theoretical framework | | | |
| Methodological orientation and Theory | 9 | What methodological orientation was stated to underpin the study? E.g., grounded theory, discourse analysis, ethnography, phenomenology, content analysis | Materials & Methods |
| Participant selection | | | |
| Sampling | 10 | How were the participants selected? E.g., purposive convenience, consecutive, snowball | Materials & Methods |
| Method of approach | 11 | How were the participants approached? E.g., face-to-face, telephone, mail, email | Materials & Methods |
| Sample size | 12 | How many participants were in the study? | Materials & Methods |
| Non-participation | 13 | How many people refused to participate or dropped out? | NA |
| Setting | | | |
| Setting of data collection | 14 | Where was the data collected? E.g., home, clinic, workplace | Materials & Methods |
| Presence of non-participants | 15 | Was anyone else present besides the participants and researchers? | NA |
| Description of sample | 16 | What are the important characteristics of the sample? E.g., demographic, data, date | Results |
| Data collection | | | |
| Interview guide | 17 | Were questions, prompts, guides provided by the authors? Was it pilot tested? | Materials & Methods |
| Repeat interviews | 18 | Were repeat interviews carried out? If yes, how many? | NA |
| Audio/visual recording | 19 | Did the research use audio or visual recording to collect the data? | Materials & Methods |
| Field notes | 20 | Were field notes made during and/or after the interview or focus group? | Materials & Methods |
| Duration | 21 | What was the duration of the interviews or focus group? | Materials & Methods |
| Data saturation | 22 | Was data saturation discussed? | Materials & Methods |
| Transcripts returned | 23 | Were transcripts returned to participants for comment and/or correction? | NA |
| **Domain 3: Analysis and findings** | | | |
| Findings | | | |
| Number of data coders | 24 | How many data coders coded the data? | Materials & Methods |
| Description of the coding tree | 25 | Did authors provide a description of the coding tree? | NA |
| Derivation of themes | 26 | Were themes identified in advance or derived from the data? | Materials & Methods |
| Software | 27 | What software, if applicable, was used to manage the data? | Materials & Methods |
| Participant checking | 28 | Did participants provide feedback on the findings? | - |
| Reporting | | | |
| Quotations presented | 29 | Were participants quotations presented to illustrate the themes/findings? Was each quotation identified? E.g., participant number? | Results |
| Data and findings consistent | 30 | Was there consistency between the data presented and the findings? | NA |
| Clarity of major themes | 31 | Were major themes clearly presented in the findings? | Results |
| Clarity of minor themes | 32 | Is there a description of diverse cases or discussion of minor themes? | Results |

Developed from: Tong A, Sainsbury P, Craig J. Consolidated criteria for reporting qualitative research (COREQ): a 32-item checklist for interviews and focus groups. International Journal for Quality in Health Care. 2007. Volume 19, Number 6: pp. 349 – 357
